# Mid-regional pro-adrenomedullin and lactate levels for risk stratification in patients with out-of-hospital cardiac arrest

**DOI:** 10.1093/ehjacc/zuad029

**Published:** 2023-03-21

**Authors:** Thomas A Zelniker, Dominik Schwall, Fardin Hamidi, Simone Steinbach, Pascal Scheller, Sebastian Spaich, Guido Michels, Evangelos Giannitsis, Hugo A Katus, Norbert Frey, Michael R Preusch

**Affiliations:** Division of Cardiology, Medical University of Vienna, Waehringer Guertel 18-20, Vienna 1090, Austria; Department of Cardiology, Angiology, and Pneumology, University Hospital Heidelberg, Im Neuenheimer Feld 410, Heidelberg 69120, Germany; Department of Cardiology, Angiology, and Pneumology, University Hospital Heidelberg, Im Neuenheimer Feld 410, Heidelberg 69120, Germany; Division of Cardiology, Medical University of Vienna, Waehringer Guertel 18-20, Vienna 1090, Austria; Department of Cardiology, Angiology, and Pneumology, University Hospital Heidelberg, Im Neuenheimer Feld 410, Heidelberg 69120, Germany; Department of Cardiology, Angiology, and Pneumology, University Hospital Heidelberg, Im Neuenheimer Feld 410, Heidelberg 69120, Germany; Department of Cardiology, Angiology, and Pneumology, University Hospital Heidelberg, Im Neuenheimer Feld 410, Heidelberg 69120, Germany; Department of Cardiology, Angiology, and Pneumology, University Hospital Heidelberg, Im Neuenheimer Feld 410, Heidelberg 69120, Germany; Department of Acute and Emergency Care, St.-Antonius-Hospital Dechant-Deckers-Straße 8, 52249 Eschweiler, Germany; Department of Cardiology, Angiology, and Pneumology, University Hospital Heidelberg, Im Neuenheimer Feld 410, Heidelberg 69120, Germany; Department of Cardiology, Angiology, and Pneumology, University Hospital Heidelberg, Im Neuenheimer Feld 410, Heidelberg 69120, Germany; DZHK (German Centre for Cardiovascular Research), Partner Site Partner Site Heidelberg/Mannheim, Heidelberg, Germany; Department of Cardiology, Angiology, and Pneumology, University Hospital Heidelberg, Im Neuenheimer Feld 410, Heidelberg 69120, Germany; DZHK (German Centre for Cardiovascular Research), Partner Site Partner Site Heidelberg/Mannheim, Heidelberg, Germany; Department of Cardiology, Angiology, and Pneumology, University Hospital Heidelberg, Im Neuenheimer Feld 410, Heidelberg 69120, Germany; DZHK (German Centre for Cardiovascular Research), Partner Site Partner Site Heidelberg/Mannheim, Heidelberg, Germany

**Keywords:** Out-of-hospital cardiac arrest, MR-proADM, Lactate, Risk stratification

## Abstract

**Aims:**

Adrenomedullin (ADM) is a free-circulating peptide that regulates endothelial barrier function and vascular tone. Here, we sought to study the relationship of ADM in combination with lactate and the risk of death in patients with out-of-hospital cardiac arrest (OHCA).

**Methods and results:**

Mid-regional pro-adrenomedullin (MR-proADM) and lactate concentrations were measured in patients with OHCA who survived at least 24 h after the return of spontaneous circulation. The outcome of interest was all-cause death. Patients were characterized by the quartiles (Q) of MR-proADM and lactate concentrations. Cox models were adjusted for age, sex, shockable rhythm, bystander resuscitation, simplified acute physiology score II (SAPS II), and estimated glomerular filtration rate (eGFR). A total of 232 patients were included in the present study (28% women, 67 years, SAPS II 80). The median MR-proADM and lactate levels at 24 h were 1.4 nmol/L [interquartile range (IQR) 0.8–2.8 nmol/L] and 1.8 mmol/L (IQR 1.3–3.4 mmol/L), respectively. Mid-regional pro-adrenomedullin concentrations correlated weakly with lactate levels (*r* = 0.36, *P* < 0.001). High (Q4) vs. low (Q1–Q3) MR-proADM concentrations were significantly associated with an increased rate of death at 28 days (75.9 vs. 45.4%; *P* < 0.001). After multivariable adjustment (including lactate levels at 24 h), higher MR-proADM levels were significantly associated with an increased risk of death [Q4 vs. Q1–Q3: adjusted hazard ratio (adj-HR) 1.67, 95% confidence interval (CI) 1.12–2.50; adj-HR for a 1-unit increase in a standardized biomarker 1.44, 95% CI 1.19–1.73]. This relationship remained significant even after further adjustment for baseline NT-proBNP and high-sensitivity troponin T levels. The combination of high MR-proADM and high lactate (Q4) concentrations identified patients at a particularly elevated risk (adj-HR 3.50; 95% CI 1.92–6.39).

**Conclusion:**

Higher MR-proADM concentrations are associated with an increased risk of death in patients with OHCA, and the combination of high MR-proADM and lactate levels identifies patients at a distinctly elevated risk.

## Graphical Abstract

**Graphical abstract:**
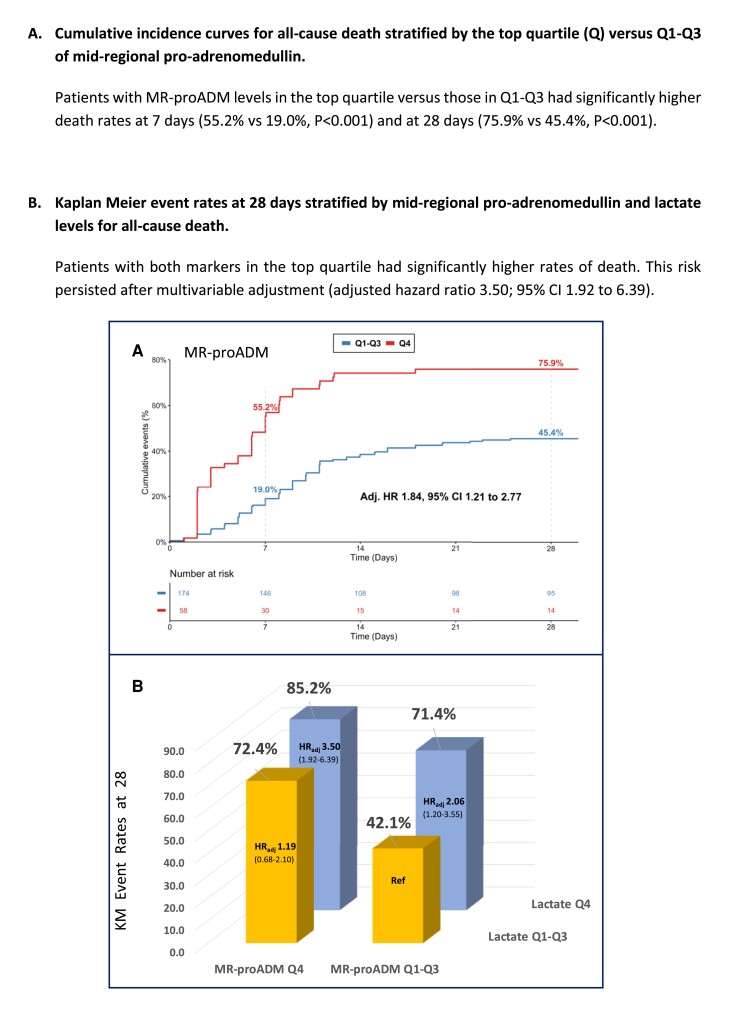
(*A*) Cumulative incidence curves for all-cause death stratified by the top quartile (Q) vs. Q1–Q3 of mid-regional pro-adrenomedullin (MR-proADM). Patients with MR-proADM levels in the top quartile vs. those in Q1–Q3 had significantly higher death rates at 7 days (55.2 vs. 19.0%, *P* < 0.001) and at 28 days (75.9 vs. 45.4%, *P* < 0.001). (*B*) Kaplan–Meier event rates at 28 days stratified by MR-proADM and lactate levels for all-cause death. Patients with both markers in the top quartile had significantly higher rates of death. This risk persisted after multivariable adjustment (adjusted hazard ratio 3.50; 95% confidence interval 1.92–6.39).

## Introduction

Despite significant advancements in intensive care medicine, managing patients resuscitated after out-of-hospital cardiac arrest (OHCA) is challenging for clinicians, and the mortality rates remain dramatically high.^1–4^ These patients frequently develop myocardial dysfunction, systemic inflammatory, and ischaemia-reperfusion response, or hypoxic brain injury, often termed *post-cardiac arrest syndrome*, leading to multiple organ failure and poor outcomes after cardiac arrest.^5,6^ Plasma biomarkers are frequently used to estimate cardiovascular risk and to aid in treatment decision-making in different populations. As a result, biomarkers are a burgeoning area of research in patients with OHCA since they offer the prospect of providing early information on the severity of organ dysfunction, allowing to make therapeutic strategy decisions, and predicting outcomes. However, despite extensive research efforts, lactate is at present the only routinely used biomarker in shock to detect hypoperfusion in patients with or without overt haemodynamic instability.^7^

Adrenomedullin (ADM) has attracted growing interest as a marker of vascular leakage and congestion in patients with sepsis and heart failure.^8–12^ ADM is a free-circulating peptide primarily synthesized by the endothelium and vascular smooth muscle cells and is responsible for regulating the endothelial barrier function and vascular tone. Intravascular ADM improves vascular integrity and decreases vascular permeability through its effects on endothelial cells, whereas in the interstitium, ADM causes vasodilatation by acting on vascular smooth muscle cells in an endothelium-independent mechanism.^10^

We hypothesized that the measurement of mid-regional pro-adrenomedullin (MR-proADM) concentrations would complement lactate and help identify patients with OHCA with vascular congestion and a damaged endothelial lining/barrier and who are at a high risk of developing post-cardiac arrest syndrome and thus of death. Here, we aimed to evaluate the relationship of MR-proADM levels in combination with lactate and death in patients with OHCA.

## Methods

The present analysis was a nested biomarker study of the ongoing prospective Heidelberg Resuscitation Registry. The Heidelberg Resuscitation Registry has been described previously.^13–16^ In brief, the Heidelberg Resuscitation Registry is a prospective registry consecutively enrolling patients after successful return of spontaneous circulation (ROSC) after non-traumatic cause of OHCA who were admitted to the intensive care unit of the University Hospital of Heidelberg. Eligible patients for this study were required to have survived at least 24 h after ROSC and to have available blood samples at 24 h. The protocol was approved by the institutional review board (S388-2011), and written informed consent was obtained from all patients or their legal representatives.

### Measurement of biomarkers

Blood samples were collected in serum-separating and EDTA-anticoagulated plastic tubes at 24 h; serum and plasma were isolated within 60 min of sample acquisition and then stored at −80°C until measurement. We measured serum levels of MR-proADM in all patients with available blood samples at 24 h using an immunofluorescent sandwich assay (Kryptor, BRAHMS, Berlin, Germany) with an analytic range extending from 0.05 to 20 nmol/L. The reported detection lower limit of detection was 0.05 nmol/L, and the reported within-run coefficient of variation was <4% at concentrations between 0.5 and 2 nmol/L. All biomarker testing was performed by personnel blinded to clinical outcomes in the affiliated research laboratories of the University Hospital Heidelberg. Moreover, concentrations of lactate were measured at both baseline and 24 h, while high-sensitivity troponin T (hsTnT) and N-terminal pro-B-type natriuretic peptide (NT-proBNP) were measured in the clinical core laboratory of the University Hospital of Heidelberg on admission.

### Outcomes of interest

The primary outcome of interest for this biomarker analysis was time to all-cause death. Specific causes of death were not adjudicated.

### Statistical analysis

We report the median observation time and the median follow-up using the reverse Kaplan–Meier estimator.^17^ The baseline patient characteristics stratified by MR-proADM quartiles were described using medians and interquartile range (IQR) for continuous variables and number and per cent for categorical variables. Spearman correlation coefficients were estimated among MR-proADM, lactate, estimated glomerular filtration rate (eGFR), NT-proBNP, and hsTnT. Kaplan–Meier event rates at 7 and 28 days were reported and compared using the log-rank test. The relationship between MR-proADM levels and death was modelled using Cox proportional hazards regression model adjusted for age, sex, the type of first monitored electrocardiogram (ECG) rhythm (shockable vs. non-shockable), presence of bystander resuscitation, simplified acute physiology score (SAPS) II, eGFR, and lactate levels. The proportional hazards assumption was confirmed using statistical tests.^18^ Mid-regional pro-adrenomedullin was modelled as a continuous standardized variable, as well as stratified by the top quartile (Q4) vs. Q1–Q3. To assess the consistency of the association between MR-proADM levels and risk of death across major subgroups (including age, eGFR, lactate levels, SAPS II, the presence of bystander resuscitation, presumed aetiology of cardiac arrest, and type of first monitored ECG rhythm), an interaction term was added to the unadjusted model. Moreover, we fitted Cox models with MR-proADM levels entered as natural splines to explore any non-linear relationship between MR-proADM levels and all-cause mortality. The degrees of freedom were selected according to the Akaike Information Criterion using the R package ‘smoothHR’.^19^ Additionally, the patient cohort was divided into four subgroups based on their MR-proADM and lactate levels: those with both markers in the top quartile, those with MR-proADM only in the top quartile, those with lactate only in the top quartile, and those with both markers in the lower three quartiles. *C*-statistics were used to assess the discriminatory value of MR-proADM and lactate levels. Statistical significance was assessed at a nominal alpha level of 0.05. All reported *P*-values are two-sided, and no adjustments for multiple testing are performed. Statistical analyses were carried out using R (version 4.1.3).^20^

## Results

### Study population

Overall, 232 patients with OHCA after successful ROSC were included. The median observation time was 18 days (IQR 7–225 days) and the median follow-up time using the reverse KM estimator was 332 days (IQR 222–514 days). The median age was 67 years (IQR 57–76 years), 66 (28%) were women, bystander cardiopulmonary resuscitation was performed in 78% of the cases, and the median time to ROSC was 20 min (IQR 11–30 min; *Table 1*). The baseline characteristics of ineligible patients are shown in Supplementary material online, *Table S1*.

**Table 1:** Baseline characteristics of the total population and stratified by quartiles of adrenomedullin levels

	Overall (*N* = 232)	Q1 (*N* = 58)	Q2 (*N* = 58)	Q3 (*N* = 58)	Q4 (*N* = 58)	*P*-value
Age	67 (57–76)	62 (49–68)	64 (54–73)	69 (61–79)	72 (61–78)	<0.001
Female sex	66 (28%)	13 (22%)	16 (28%)	22 (38%)	15 (26%)	0.28
Bystander CPR	180 (78%)	47 (81%)	45 (79%)	48 (83%)	40 (69%)	0.28
Time to ROSC (min)	20 (11–30)	18 (10–25)	20 (12–24)	21 (11–38)	23 (13–32)	0.18
Shockable rhythm	137 (59%)	38 (66%)	41 (71%)	36 (62%)	22 (38%)	0.001
Cause of resuscitation of presumed cardiovascular origin	173 (75%)	42 (72%)	48 (83%)	46 (79%)	37 (64%)	0.10
Diabetes	50 (22%)	5 (9%)	11 (19%)	13 (22%)	21 (36%)	0.004
eGFR (mL/min/1.73 m^2^)	63 (48–82)	79 (57–94)	76 (59–88)	61 (45–70)	47 (36–63)	<0.001
Lactate (mmol/L) upon admission	4.8 (2.8–7.4)	4.5 (2.4–6.6)	3.5 (1.9–6.1)	5.2 (3.5–7.3)	6.1 (3.7–10.2)	<0.001
Lactate >2.0 mmol/L upon admission	190 (86%)	44 (80%)	40 (71%)	53 (95%)	53 (96%)	<0.001
SAPS II	80 (72–89)	74 (63–82)	76 (68–79)	84 (76–91)	88 (82–96)	<0.001

Continuous variables are reported as medians (25th to 75th percentile).

CPR, cardiopulmonary resuscitation; eGFR, estimated glomerular filtration rate; ROSC, return of spontaneous circulation, SAPS II, simplified acute physiology score II.

One hundred and thirty-seven (59%) patients in the study presented with an initial shockable rhythm. Of the 173 (75%) patients who had a cardiac arrest of cardiovascular origin, ischaemic heart disease was the presumed cause for 124 patients (72%), of which 48 patients (27.4%) had a diagnosis of ST-elevation myocardial infarction, and 6 patients (3.5%) were diagnosed with a pulmonary embolism. The median SAPS II was 80 (IQR 72–89), and the median lactate levels were 4.8 mmol/L (IQR 2.8–7.4 mmol/L) upon admission and 1.8 mmol/L (1.3–3.4 mmol/L) at 24 h. In total, 132 patients (56.9%) died and 24 patients had an unfavourable neurological outcome (i.e. a cerebral performance category score of 3–4).

### Mid-regional pro-adrenomedullin concentrations at 24 h after out-of-hospital cardiac arrest

The median MR-proADM levels at 24 h were 1.4 nmol/L (IQR 0.8–2.8 nmol/L). Patients with higher MR-proADM quartiles were more likely to be older, have diabetes, a lower eGFR, higher lactate concentrations and SAPS II, and less likely to have a shockable rhythm (*Table 1*).

Mid-regional pro-adrenomedullin significantly correlated with eGFR at baseline (*r* = −0.42, *P* < 0.001) and at 24 h (*r* = −0.56, *P* < 0.001), with baseline eGFR available in all patients and 24 h eGFR available in 225/232 (97.0%) patients, as well as with lactate levels at baseline (*r* = 0.23, *P* < 0.001) and at 24 h (*r* = 0.36, *P* < 0.001). Mid-regional pro-adrenomedullin was also significantly correlated with NT-proBNP (*r* = 0.43, *P* < 0.001) but not with hsTnT (*r* = −0.09, *P* = 0.21) levels on admission that were available in 173 (75%) and 218 (94.0%) patients, respectively. Moreover, MR-proADM significantly correlated with the SAPS II (*r* = 0.48, *P* < 0.001).

### Relationship between mid-regional pro-adrenomedullin and death in patients with out-of-hospital cardiac arrest

**Figure 1:**
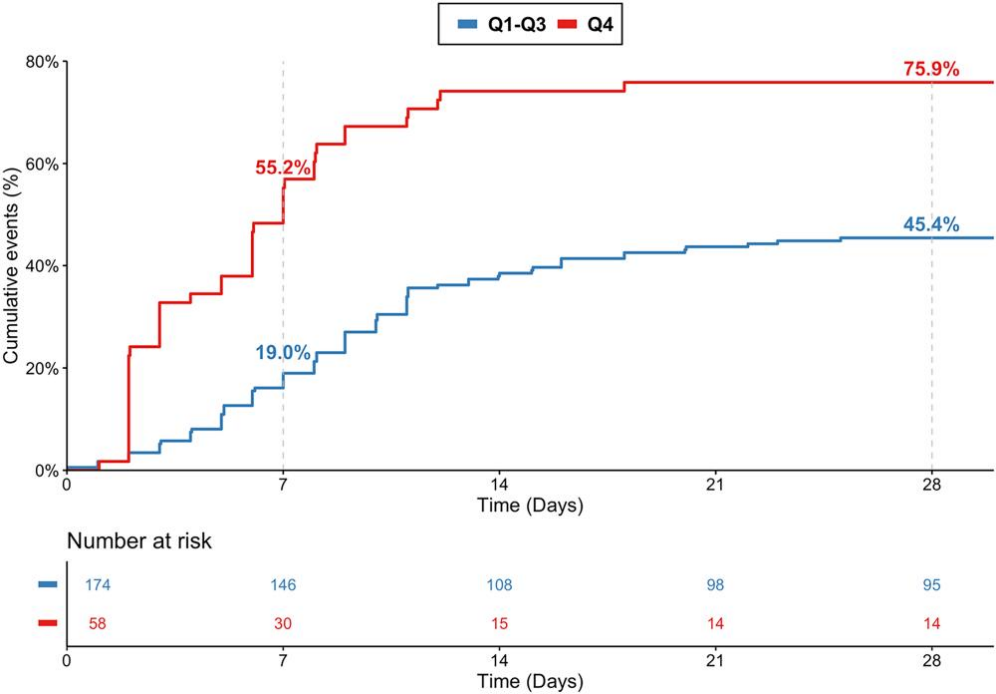
Cumulative incidence curve with the respective Kaplan–Meier event rates at 7 and 28 days for all-cause death stratified by the top quartile (Q4) vs. Q1–Q3 of mid-regional pro-adrenomedullin levels.

**Figure 2:**
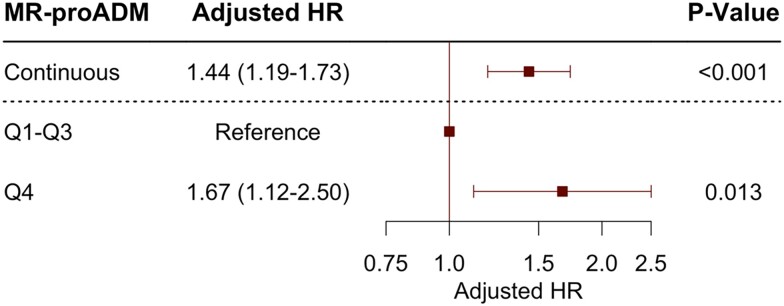
Adjusted hazard ratios for mid-regional pro-adrenomedullin concentrations modelled as a continuous variable per 1 standard deviation increase in biomarker and stratified by the top quartile (Q4) vs. Q1–Q3 of adrenomedullin levels for all-cause death.

Patients with MR-proADM levels in the top quartile vs. those in Q1–Q3 had significantly higher death rates at 7 days (55.2 vs. 19.0%, *P* < 0.001) and at 28 days (75.9 vs. 45.4%, *P* < 0.001; *Figure 1*). Mid-regional pro-adrenomedullin concentrations in Q4 vs. Q1–Q3 remained independently associated with an increased risk of death after multivariable adjustment for age, sex, shockable rhythm, bystander resuscitation, SAPS II, eGFR, and lactate levels [adjusted hazard ratio (adj-HR) 1.67, 95% confidence interval (CI) 1.12–2.50]. The relationship between MR-proADM levels and death was also statistically significant when MR-proADM was modelled as a continuous variable (adj-HR for 1 unit increase in standardized biomarker 1.44, 95% CI 1.19–1.73; *Figure 2*). This association remained significant in sensitivity analyses after further adjustment for time to ROSC (adj-HR for 1 unit increase in standardized biomarker 1.43, 95% CI 1.16–1.79; adjusted HR for Q4 vs. Q1–Q3: 1.63, 95% CI 1.03–2.59), as well as for NT-proBNP and hsTnT (adjusted HR for 1 unit increase in standardized biomarker 1.47, 95% CI 1.15–1.88; adjusted HR for Q4 vs. Q1–Q3 2.00, 95% CI 1.15–3.50).

**Figure 3:**
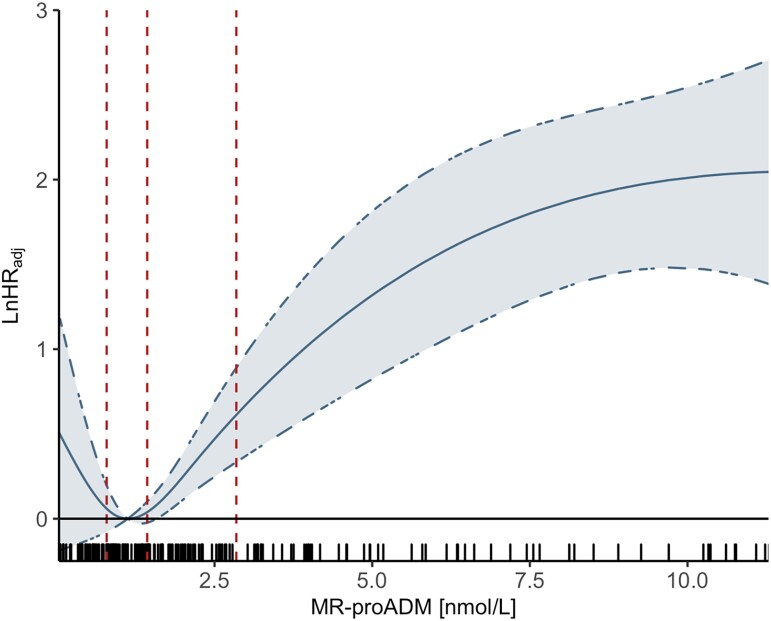
Estimated adjusted log-hazard ratios and 95% confidence limits for all-cause death in relation to continuous mid-regional pro-adrenomedullin concentrations modelled using natural cubic splines. The dashed vertical lines indicate the 25th, 50th, and 75th percentiles. The rug plot illustrates the marginal distribution of adrenomedullin concentrations. Cox regression models were adjusted for age, sex, the type of first monitored electrocardiogram rhythm, presence of bystander resuscitation, estimated glomerular filtration rate, and lactate levels.

Continuous MR-proADM, modelled with adjusted natural cubic regression splines, indicated a consistent increase in the hazard of death from the median of MR-proADM concentrations (*Figure 3*).

### Subgroup analyses

The association between MR-proADM levels and the risk of death was consistent across different tested subgroups, including age, eGFR, lactate levels, SAPS II, the presence of bystander resuscitation, presumed aetiology of cardiac arrest, and type of first monitored ECG rhythm (all *P*-interaction >0.09). However, the relationship between MR-proADM concentrations and death tended to be stronger in men (HR 2.53, 95% CI 1.83–3.48) than in women (HR 1.72, 95% CI 1.46–2.02; *P-*value for interaction 0.031).

### Concurrent stratification by mid-regional pro-adrenomedullin and lactate

**Figure 4:**
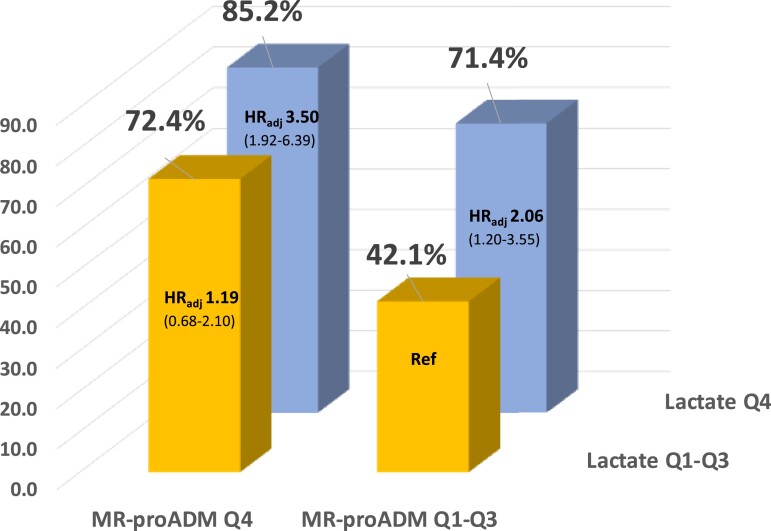
Kaplan–Meier event rates at 28 days and adjusted hazard ratios for all-cause death stratified by mid-regional pro-adrenomedullin and lactate concentrations.

Lactate levels both measured on admission (adjusted HR for Q4 vs. Q1–Q3 1.80; 95% CI 1.22–2.66) and at 24 h (adjusted HR for Q4 vs. Q1–Q3 2.24; 95% CI 1.47–3.42) were significantly associated with death. When patients were stratified simultaneously by both MR-proADM and lactate quartiles measured at 24 h, patients with both markers in Q4 had significantly higher rates of death compared with patients with both markers in Q1–Q3 (85.2 vs. 42.1%, *P* < 0.001; *Figure 4*). This risk persisted after multivariable adjustment (adjusted HR 3.50; 95% CI 1.92–6.39). Of note, patients with MR-proADM levels in the top quartile but lactate levels in Q1–Q3 had similar event rates (71.4 and 72.4%, *P* = 0.80) compared with patients with lactate levels in Q4 and MR-proADM levels in Q1–Q3 though the adjusted hazard of death was higher in patients with lactate levels in Q4.

### Discriminatory performance of mid-regional pro-adrenomedullin and lactate

The discriminatory performance for death as indicated by the area under the curve (AUC) of MR-proADM, lactate, and the combination of both biomarkers was 0.69 (95% CI 0.62–0.75), 0.62 (95% 0.55–0.70), and 0.77 (95% CI 0.71–0.83), respectively. Mid-regional pro-adrenomedullin performed similarly well (*P* = 0.10) compared with the SAPS II (AUC 0.75, 95% CI 0.69–0.82), and adding MR-proADM to the baseline SAPS II significantly increased the AUC (Δ = 0.025, *P* = 0.046).

## Discussion

In the present biomarker study from the Heidelberg Resuscitation Registry, including 232 patients with OHCA of non-traumatic origin who survived at least 24 h after ROSC, we found that MR-proADM serum levels were significantly associated with the risk of death after multivariable adjustment, including traditional cardiovascular biomarkers. Moreover, the combination of high MR-proADM and high lactate levels identified patients at a distinctly elevated risk.

### Unmet needs in risk stratification of patients with out-of-hospital cardiac arrest

There are many different aetiologies for OHCA, resulting in patients with OHCA often representing a heterogeneous population with various underlying comorbidities and complex needs. Moreover, many patients with prolonged delay in time to resuscitation have a poor prognosis due to irreversible cerebral hypoxia irrespective of subsequent treatment followed. As a result, risk stratification of patients with OHCA is exceedingly complex. However, early identification of patients who develop shock due to myocardial dysfunction, systemic ischaemia-reperfusion or inflammatory response, or sepsis is critical. Previous data indicate that patients with OHCA who die from haemodynamic deterioration have distinct admission biomarker profiles compared with those who have cerebral anoxia.^21^ The early separation of the Kaplan–Meier curves suggests that MR-proADM could be a useful biomarker for identifying haemodynamic stress in resuscitated patients with OHCA. Several clinical risk scores have been proposed but are currently not recommended to form the main foundation of clinical decision-making and are therefore, infrequently used in clinical practice.^22–24^

### Adrenomedullin in out-of-hospital cardiac arrest

In healthy individuals, ADM circulates at low levels. Aside from the profound vasodilatory effects that ADM exerts through its effects on vascular smooth muscle cells, ADM also contributes to preserving endothelial integrity. Vasodilation and vascular leakage caused by disrupted endothelial integrity play a pivotal role in developing a post-cardiac arrest syndrome or shock in general. Interestingly, in a Danish cohort study, higher MR-proADM concentrations were measured in patients with cardiogenic shock related to acute myocardial infarction than in patients with acute myocardial infarction and OHCA.^25^ These findings imply that cardiogenic shock due to a low cardiac output varies from that caused by cardiac arrest characterized by an abrupt and complete cessation of circulation. The present study thus expands these findings, indicating that MR-proADM concentrations can identify individuals with OHCA who develop complications of post-cardiac arrest syndrome.

### Combined value of lactate and adrenomedullin

In addition, we observed that MR-proADM provided complementary information on risk stratification in addition to lactate levels. Lactate is a well-established surrogate marker for adequate tissue perfusion and is used for identifying and monitoring patients with sepsis^26^ and cardiogenic shock.^7,26^ However, although the SCAI SHOCK stages that include lactate correlate well with mortality,^7^ lactate levels measured as early as on admission have been found to perform only moderately well as an individual biomarker for predicting death in patients with OHCA.^27–29^ Our findings, however, suggest that a multimarker approach combining MR-proADM and lactate levels could help assess and identify patients at distinctly elevated risk of death.

### Sex-specific differences in adrenomedullin levels in patients with out-of-hospital cardiac arrest

Although MR-proADM concentrations and death were significantly associated in both men and women, we observed that the relationship tended to be stronger in men than in women. Recent studies have indicated a complex interplay between sex hormones, angiotensin II, and the production and vasodilatory effects of ADM.^30,31^ Oestradiol has been shown to reduce the angiotensin-II-induced increase in the number of ADM-secreting endothelial cells^31^ while increasing the ADM-induced vasodilation.^30^ These data thus add to the mounting body of evidence of sex-based differences in outcomes after OHCA, including lower rates of a first shockable rhythm and lower survival rates in women.^32,33^

### Adrenomedullin as a potential treatment target

The increasing evidence indicating the protective properties of ADM on reducing endothelial permeability and improving endothelial stability and microcirculation, thereby reducing organ damage, as well as its anti-microbial activity, has prompted several clinical trials in sepsis and heart failure to investigate the potential therapeutic use of antibodies, such as adrecizumab targeting ADM. Because of their large molecular weight, these antibodies cannot pass through the endothelium barrier and thus remain in circulation, where they have no adverse vasodilatory effects. The current findings may suggest that adrecizumab be studied in patients with OHCA. Future research should also address whether MR-proADM levels can be utilized to guide diuretic therapy, monitor disease progression, or evaluate the efficacy of therapeutic approaches.

### Limitations

Several limitations, including the single-centre setting and its exploratory nature, should be addressed. The findings of this study cannot be generalized to all patients with OHCA as only those who survived the first 24 h were included. Despite multivariable adjustment for a broad group of relevant covariates, potential residual confounding may exist in our findings. Although treating physicians were unaware of the MR-proADM concentrations, they were aware of the lactate levels. Furthermore, we did not evaluate the specific causes of death, and the lack of serial biomarker measurements prevents us from drawing any conclusions about kinetics. Moreover, whether new immunoassays that measure the biologically active ADM may perform even better in this cohort remains unclear. Due to the exploratory nature of this analysis, no adjustments for multiple testing were performed.

## Conclusions

Higher MR-proADM concentrations are associated with an increased risk of death in patients with OHCA of non-traumatic origin, and the combination of high MR-proADM and high lactate levels identifies patients at a distinctly elevated risk.

## Supplementary material

Supplementary material is available at *European Heart Journal: Acute Cardiovascular Care* online.

## Supplementary Material

zuad029_Supplementary_Data

## Data Availability

The data, analytic methods, and study materials will not be made available to other researchers for the purposes of reproducing the results or replicating the procedure. However, we encourage parties interested in collaboration and data sharing to contact the corresponding authors directly for further discussions.
