# Supplementary material for: Mid-regional pro-adrenomedullin and lactate levels for risk stratification in patients with out-of-hospital cardiac arrest
Source: Eur Heart J Acute Cardiovasc Care. 2023 Mar 21;12(6):364–71. doi: 10.1093/ehjacc/zuad029 (PMC10236520; doi:10.1093/ehjacc/zuad029)
Supplement: zuad029_Supplementary_Data [file zuad029_supplementary_data.docx]

**Appendix**

**Supplemental Table 1: Baseline characteristics of ineligible patients**

| **Characteristic** | **N = 43** |
| --- | --- |
| **Age** | 66 (57, 76) |
| **Female Sex** | 0 (0%) |
| **Bystander CPR** | 33 (77%) |
| **Time to ROSC (min)** | 21 (15, 30) |
| **Shockable rhythm** | 0 (0%) |
| **eGFR upon admission (ml/min/1.73m2)** | 61 (49, 78) |
| **Lactate upon admission (mmol/L)** | 5.5 (3.5, 7.7) |
| **Lactate upon admission >2.0 mmol/L** | 39 (91%) |
| **SAPS II score** | 67 (62, 75) |
|  |  |
